# 1-(3-Acetyl­phen­yl)-2-(2-oxidonaph­thalen-1-yl)diazen-1-ium

**DOI:** 10.1107/S1600536813014918

**Published:** 2013-06-08

**Authors:** Hassiba Bougueria, Ali Benosmane, Mohamed Amine Benaouida, Abd El Kader Bouchoul, Salah Eddine Bouaoud

**Affiliations:** a Unité de Recherche de Chimie de l’Environnement et Moléculaire Structurale (CHEMS), Département de Chimie, Université Mentouri de Constantine 1, 25000 Constantine, Algeria

## Abstract

The title compound, C_18_H_14_N_2_O_2_, crystallized with two independent zwitterion mol­ecules (*A* and *B*) in the asymmetric unit. They are both close to planar, the dihedral angle between the benzene ring and naphthalene ring system being 4.30 (9)° in *A* and 4.69 (9)° in *B*. Each mol­ecule has an *E* conformation with respect to the azo double bond. In each of the independent mol­ecules, an intra­molecular N—H⋯O hydrogen bond forms an *S*(6) ring motif. In the crystal, mol­ecules are linked *via* C—H⋯O hydrogen bonds, forming –*A*—*A*—*A*– and –*B*—*B*—*B*– chains parallel to one another and propagating along the *a*-axis direction. There are also π–π inter­actions between adjacent mol­ecules involving benzene and naphthalene rings [centroid–centroid distance of 3.626 (3) Å for adjacent *A* mol­ecules and 3.652 (3) Å for adjacent *B* mol­ecules].

## Related literature

For general background to azo compounds and their use in dyes, pigments and advanced materials, see: Lee *et al.* (2004 ▶); Oueslati *et al.* (2004 ▶). Many azo compounds have been synthesized by diazo­tization and diazo coupling reactions, see: Wang *et al.* (2003 ▶). For a related structure, see: Rãdulescu *et al.* (2006 ▶).
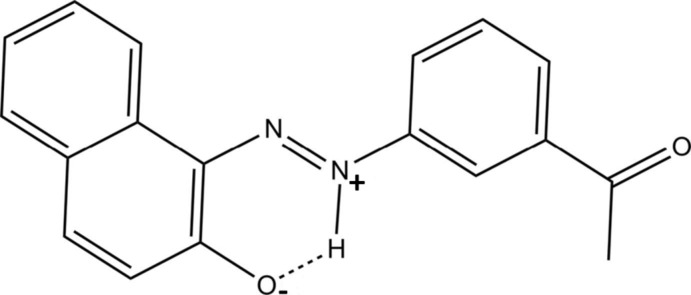


## Experimental

### 

#### Crystal data

C_18_H_14_N_2_O_2_
*M* *_r_* = 290.31
Orthorhombic, 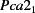

*a* = 15.965 (5) Å
*b* = 5.807 (5) Å
*c* = 30.185 (5) Å
*V* = 2798 (3) Å^3^
*Z* = 8
Mo *K*α radiation
μ = 0.09 mm^−1^
*T* = 150 K
0.26 × 0.22 × 0.17 mm

#### Data collection

Bruker APEXII diffractometer
Absorption correction: multi-scan (*SADABS*; Bruker, 2006 ▶) *T*_min_ = 0.830, *T*_max_ = 0.985
13123 measured reflections
5097 independent reflections
4621 reflections with *I* > 2σ(*I*)
*R*_int_ = 0.028

#### Refinement

*R*[*F* ^2^ > 2σ(*F* ^2^)] = 0.043
*wR*(*F* ^2^) = 0.120
*S* = 1.03
5097 reflections
399 parameters
1 restraint
H-atom parameters constrained
Δρ_max_ = 0.24 e Å^−3^
Δρ_min_ = −0.22 e Å^−3^

### 

Data collection: *APEX2* (Bruker, 2006 ▶); cell refinement: *SAINT* (Bruker, 2006 ▶); data reduction: *SAINT*; program(s) used to solve structure: *SIR97* (Altomare *et al.*, 1999 ▶); program(s) used to refine structure: *SHELXL97* (Sheldrick, 2008 ▶); molecular graphics: *PLATON* (Spek, 2009 ▶); software used to prepare material for publication: *WinGX* (Farrugia, 2012 ▶).

## Supplementary Material

Crystal structure: contains datablock(s) global, I. DOI: 10.1107/S1600536813014918/su2604sup1.cif

Structure factors: contains datablock(s) I. DOI: 10.1107/S1600536813014918/su2604Isup2.hkl

Supplementary material file. DOI: 10.1107/S1600536813014918/su2604Isup3.cml

Additional supplementary materials:  crystallographic information; 3D view; checkCIF report

## Figures and Tables

**Table 1:** Hydrogen-bond geometry (Å, °)

*D*—H⋯*A*	*D*—H	H⋯*A*	*D*⋯*A*	*D*—H⋯*A*
N13—H13⋯O1	0.86	1.91	2.580 (3)	134
N43—H43⋯O31	0.86	1.90	2.575 (3)	134
C15—H15⋯O22^i^	0.93	2.36	3.256 (4)	162
C45—H45⋯O52^ii^	0.93	2.36	3.256 (4)	162

Symmetry codes: (i) 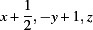; (ii) 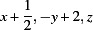.

**Fig. 1.**
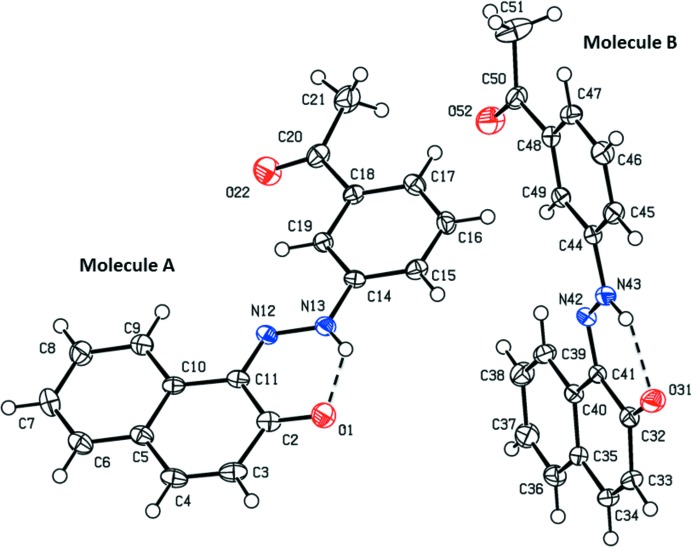
A view of the molecular structure of the two independent molecules (A and B) of the title compound, with atom labelling. Displacement ellipsoids are drawn at the 50% probability level. The intramolecular N—H···O hydrogen bonds are shown as dashed lines (see Table 1 for details).

**Fig. 2.**
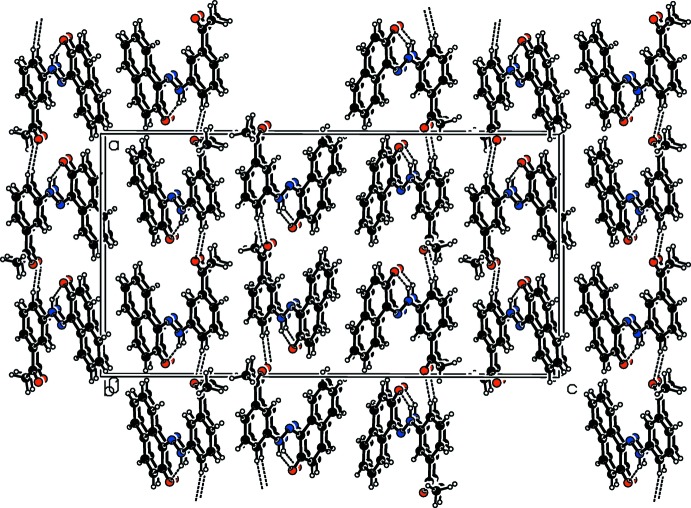
A view along the b axis of the crystal packing of the title compound. The N—H···O and C—H···O hydrogen bonds are shown as dashed lines (see Table 1 for details).

## supplementary crystallographic information

### Comment

The azo dyes are by far the most important clas of dyes, accounting for over 50% of all commercial dyes, and having been studied more than any other class. Azo dyes contain at least one azo group (–N=N–) but can contain two (diazo), three(triazo), or more rarely, four (tetrakisazo) or more (polyazo). The azo group is attached to two groups, of which at least one, but more usually both are aromatic. They exist in the *trans* form with the bond angle vis.120°, about the sp2 hybridized N atoms. Almost without exception, azo dyes are made by diazotization of a primary aromatic amine followed by coupling of the resultant diazonium salt with an electron-rich nucleophile (Wang *et al.*, 2003). We report herein on the crystal structure of the title compound, obtained through the diazotization of 3-acetoaniline followed by a coupling reaction with 2-naphthol.

The molecular structures of the two independent molecules (A and B) of the title compound are shown in Fig. 1. In both molecules the hydrogen atom of the OH group has been transfered to the N atom in the azo group to form a zwitterion. There are no significant differences in their structures. The dihedral angle between the benzene ring and naphthalene ring system is 4.30 (9) ° in A and 4.69 (9) ° in the B. Each molecule has an E conformation with respect to the azo double bond. The torsion angle C11—N12—N13—C14 being 179.7 (2) ° in A, while in B the corresponding torsion angle C41–N42–N43–C44 is 179.2 (2)°. An intramolecular N—H···O hydrogen bond exists in each molecule (Table 1), forming an S(6) ring motif.

In the crystal, molecules are linked *via* C—H···O hydrogen bonds forming –A—A—A– and –B—B—B– chains parallel to one another and propagating along the *a* axis direction. There are also π-π interactions between adjacent molecules involving benzene and naphthalene rings: *Cg*1···*Cg*3^i^ = 3.626 (3) Å for adjacent A molecules [*Cg*1 and *Cg*3 are the centroids of the C2—C5/C10/C11 and C5—C10 rings; symmetry code: (i) *x*, *y* - 1, *z*] and *Cg*5···*Cg*7^i^ = 3.652 (3) Å for adjacent B molecules [*Cg*5 and *Cg*7 are the centroids of the C32—C35/C40/C41 and C44—C49 rings; symmetry code: (i) *x*, *y* - 1, *z*].

### Experimental

The title compound was synthesized according to the literature procedure used for the synthesis of other aromatic azo-compounds (Wang *et al.*, 2003). Red prisms of the compound were obtained by slow evaporation at room temperature of a H_2_O/THF (1/1 *v*/*v*) solution of the title compound.

### Refinement

The NH H atoms were located in difference Fourier maps. In the final cycles of refinement they and the C-bound H atoms were included in calculated positions and treated as riding atoms: N—H = 0.86 Å, C—H = 0.93 and 0.96 Å for CH and CH_3_ H atoms, respectively, with *U*_iso_(H)= 1.5*U*_eq_(*C*-methyl) and = 1.2*U*_eq_(N,*C*) for other H atoms.

### Crystal data

**Table:**

C_18_H_14_N_2_O_2_	*F*(000) = 1216
*M**_r_* = 290.31	*D*_x_ = 1.378 Mg m^−^^3^
Orthorhombic, *P**c**a*2_1_	Mo *K*α radiation, λ = 0.71073 Å
Hall symbol: P 2c -2ac	Cell parameters from 8593 reflections
*a* = 15.965 (5) Å	θ = 2.7–27.5°
*b* = 5.807 (5) Å	µ = 0.09 mm^−^^1^
*c* = 30.185 (5) Å	*T* = 150 K
*V* = 2798 (3) Å^3^	Prism, red
*Z* = 8	0.26 × 0.22 × 0.17 mm

### Data collection

**Table:**

Bruker APEXII diffractometer	5097 independent reflections
Radiation source: fine-focus sealed tube	4621 reflections with *I* > 2σ(*I*)
Graphite monochromator	*R*_int_ = 0.028
CCD rotation images, thin slices scans	θ_max_ = 27.5°, θ_min_ = 2.9°
Absorption correction: multi-scan (*SADABS*; Bruker, 2006)	*h* = −20→17
*T*_min_ = 0.830, *T*_max_ = 0.985	*k* = −6→7
13123 measured reflections	*l* = −39→23

### Refinement

**Table:**

Refinement on *F*^2^	Primary atom site location: structure-invariant direct methods
Least-squares matrix: full	Secondary atom site location: difference Fourier map
*R*[*F*^2^ > 2σ(*F*^2^)] = 0.043	Hydrogen site location: inferred from neighbouring sites
*wR*(*F*^2^) = 0.120	H-atom parameters constrained
*S* = 1.03	*w* = 1/[σ^2^(*F*_o_^2^) + (0.076*P*)^2^ + 0.6139*P*] where *P* = (*F*_o_^2^ + 2*F*_c_^2^)/3
5097 reflections	(Δ/σ)_max_ < 0.001
399 parameters	Δρ_max_ = 0.24 e Å^−^^3^
1 restraint	Δρ_min_ = −0.22 e Å^−^^3^

### Special details

**Table:**

Geometry. Bond distances, angles *etc*. have been calculated using the rounded fractional coordinates. All su's are estimated from the variances of the (full) variance-covariance matrix. The cell e.s.d.'s are taken into account in the estimation of distances, angles and torsion angles
Refinement. Refinement on *F*^2^ for ALL reflections except those flagged by the user for potential systematic errors. Weighted *R*-factors *wR* and all goodnesses of fit *S* are based on *F*^2^, conventional *R*-factors *R* are based on *F*, with *F* set to zero for negative *F*^2^. The observed criterion of *F*^2^ > σ(*F*^2^) is used only for calculating -*R*-factor-obs *etc*. and is not relevant to the choice of reflections for refinement. *R*-factors based on *F*^2^ are statistically about twice as large as those based on *F*, and *R*-factors based on ALL data will be even larger.

### Fractional atomic coordinates and isotropic or equivalent isotropic displacement parameters (Å^2^)

**Table:**

	*x*	*y*	*z*	*U*_iso_*/*U*_eq_	
O1	0.88455 (10)	0.0348 (3)	0.91871 (6)	0.0302 (5)	
O22	0.46944 (10)	0.5866 (3)	0.85810 (8)	0.0412 (7)	
N12	0.71060 (12)	0.1448 (3)	0.91349 (6)	0.0196 (5)	
N13	0.76073 (11)	0.2825 (3)	0.89137 (6)	0.0205 (5)	
C2	0.83087 (14)	−0.0922 (4)	0.93640 (8)	0.0221 (6)	
C3	0.85443 (15)	−0.3073 (4)	0.95777 (8)	0.0257 (7)	
C4	0.79693 (15)	−0.4408 (4)	0.97746 (9)	0.0244 (7)	
C5	0.70855 (14)	−0.3830 (4)	0.97893 (7)	0.0214 (6)	
C6	0.65146 (16)	−0.5256 (4)	1.00018 (8)	0.0266 (7)	
C7	0.56723 (16)	−0.4703 (4)	1.00171 (9)	0.0296 (7)	
C8	0.53960 (15)	−0.2689 (4)	0.98130 (9)	0.0279 (7)	
C9	0.59476 (15)	−0.1250 (4)	0.95991 (8)	0.0237 (6)	
C10	0.68068 (14)	−0.1781 (4)	0.95840 (7)	0.0186 (6)	
C11	0.74111 (14)	−0.0333 (4)	0.93559 (8)	0.0199 (6)	
C14	0.72637 (14)	0.4698 (4)	0.86804 (8)	0.0183 (6)	
C15	0.78104 (13)	0.6187 (4)	0.84599 (8)	0.0214 (6)	
C16	0.74934 (15)	0.8061 (4)	0.82347 (8)	0.0228 (7)	
C17	0.66327 (14)	0.8496 (4)	0.82345 (8)	0.0224 (6)	
C18	0.60908 (13)	0.7014 (4)	0.84491 (8)	0.0191 (6)	
C19	0.64032 (13)	0.5082 (4)	0.86734 (8)	0.0195 (6)	
C20	0.51564 (14)	0.7373 (4)	0.84465 (8)	0.0251 (6)	
C21	0.48199 (16)	0.9589 (5)	0.82685 (10)	0.0382 (9)	
O31	0.93956 (10)	0.5296 (3)	0.64568 (7)	0.0307 (5)	
O52	0.52562 (10)	1.0856 (3)	0.70523 (8)	0.0406 (6)	
N42	0.76561 (11)	0.6405 (3)	0.65143 (7)	0.0201 (5)	
N43	0.81623 (11)	0.7766 (3)	0.67337 (6)	0.0214 (5)	
C32	0.88584 (14)	0.4026 (4)	0.62827 (8)	0.0229 (6)	
C33	0.90840 (15)	0.1882 (4)	0.60672 (8)	0.0254 (7)	
C34	0.85114 (15)	0.0550 (4)	0.58701 (8)	0.0258 (7)	
C35	0.76317 (15)	0.1131 (4)	0.58568 (8)	0.0213 (6)	
C36	0.70485 (16)	−0.0288 (4)	0.56436 (9)	0.0277 (7)	
C37	0.62120 (17)	0.0288 (5)	0.56317 (9)	0.0304 (8)	
C38	0.59387 (15)	0.2310 (4)	0.58362 (9)	0.0290 (7)	
C39	0.64969 (14)	0.3723 (4)	0.60486 (8)	0.0238 (7)	
C40	0.73576 (14)	0.3193 (4)	0.60642 (8)	0.0210 (6)	
C41	0.79672 (14)	0.4629 (4)	0.62934 (8)	0.0192 (6)	
C44	0.78179 (14)	0.9636 (4)	0.69705 (7)	0.0194 (6)	
C45	0.83625 (14)	1.1076 (4)	0.72006 (8)	0.0226 (6)	
C46	0.80436 (15)	1.2942 (4)	0.74318 (8)	0.0244 (7)	
C47	0.71858 (15)	1.3399 (4)	0.74312 (8)	0.0236 (7)	
C48	0.66479 (14)	1.1958 (4)	0.71987 (7)	0.0208 (6)	
C49	0.69584 (13)	1.0051 (4)	0.69702 (8)	0.0202 (6)	
C50	0.57227 (15)	1.2340 (4)	0.71913 (9)	0.0258 (7)	
C51	0.53792 (18)	1.4575 (5)	0.73668 (11)	0.0427 (9)	
H3	0.91030	−0.35290	0.95780	0.0310*	
H4	0.81450	−0.57680	0.99080	0.0290*	
H6	0.67010	−0.66030	1.01360	0.0320*	
H7	0.52950	−0.56640	1.01620	0.0360*	
H8	0.48300	−0.23120	0.98220	0.0330*	
H9	0.57510	0.00840	0.94640	0.0280*	
H13	0.81390	0.25850	0.89120	0.0250*	
H15	0.83840	0.59160	0.84650	0.0260*	
H16	0.78540	0.90390	0.80830	0.0270*	
H17	0.64240	0.97870	0.80890	0.0270*	
H19	0.60400	0.40710	0.88160	0.0230*	
H21A	0.42250	0.96430	0.83130	0.0570*	
H21B	0.49410	0.96930	0.79580	0.0570*	
H21C	0.50780	1.08560	0.84210	0.0570*	
H33	0.96420	0.14220	0.60660	0.0300*	
H34	0.86870	−0.08090	0.57360	0.0310*	
H36	0.72290	−0.16390	0.55080	0.0330*	
H37	0.58310	−0.06660	0.54880	0.0370*	
H38	0.53740	0.27010	0.58280	0.0350*	
H39	0.63040	0.50570	0.61850	0.0290*	
H43	0.86940	0.75250	0.67330	0.0260*	
H45	0.89360	1.07890	0.71990	0.0270*	
H46	0.84050	1.38980	0.75890	0.0290*	
H47	0.69750	1.46590	0.75850	0.0280*	
H49	0.65960	0.90700	0.68200	0.0240*	
H51A	0.47800	1.45710	0.73420	0.0640*	
H51B	0.56040	1.58330	0.71980	0.0640*	
H51C	0.55350	1.47470	0.76720	0.0640*	

### Atomic displacement parameters (Å^2^)

**Table:**

	*U*^11^	*U*^22^	*U*^33^	*U*^12^	*U*^13^	*U*^23^
O1	0.0210 (8)	0.0295 (9)	0.0401 (10)	−0.0016 (7)	−0.0002 (8)	0.0022 (8)
O22	0.0175 (8)	0.0353 (10)	0.0707 (15)	−0.0053 (7)	−0.0001 (9)	0.0051 (10)
N12	0.0225 (9)	0.0152 (8)	0.0211 (9)	−0.0013 (7)	−0.0015 (8)	−0.0018 (7)
N13	0.0177 (9)	0.0172 (8)	0.0266 (10)	−0.0001 (7)	0.0003 (8)	0.0001 (8)
C2	0.0202 (11)	0.0211 (10)	0.0250 (12)	0.0014 (9)	−0.0047 (9)	−0.0037 (10)
C3	0.0225 (11)	0.0243 (11)	0.0304 (13)	0.0067 (9)	−0.0079 (10)	−0.0060 (10)
C4	0.0276 (12)	0.0204 (11)	0.0252 (13)	0.0072 (10)	−0.0093 (10)	−0.0006 (9)
C5	0.0285 (12)	0.0176 (10)	0.0181 (11)	0.0010 (9)	−0.0042 (9)	−0.0027 (9)
C6	0.0331 (13)	0.0217 (11)	0.0251 (12)	0.0008 (10)	−0.0053 (10)	0.0023 (10)
C7	0.0312 (13)	0.0289 (12)	0.0287 (13)	−0.0090 (10)	−0.0001 (10)	0.0058 (11)
C8	0.0211 (10)	0.0292 (11)	0.0333 (13)	−0.0021 (10)	−0.0004 (10)	0.0014 (10)
C9	0.0252 (11)	0.0216 (10)	0.0243 (11)	0.0017 (9)	−0.0004 (9)	−0.0004 (10)
C10	0.0212 (11)	0.0171 (10)	0.0175 (10)	0.0024 (9)	−0.0032 (9)	−0.0044 (9)
C11	0.0223 (10)	0.0162 (10)	0.0212 (11)	0.0022 (8)	−0.0025 (9)	−0.0031 (9)
C14	0.0197 (11)	0.0153 (9)	0.0198 (11)	0.0009 (8)	−0.0024 (9)	−0.0031 (9)
C15	0.0158 (10)	0.0228 (10)	0.0255 (11)	−0.0005 (8)	−0.0010 (9)	−0.0045 (9)
C16	0.0229 (11)	0.0212 (11)	0.0243 (12)	−0.0050 (9)	0.0019 (10)	0.0012 (10)
C17	0.0268 (11)	0.0189 (10)	0.0215 (11)	−0.0013 (9)	−0.0030 (10)	−0.0001 (9)
C18	0.0140 (10)	0.0217 (10)	0.0216 (11)	0.0012 (8)	−0.0016 (9)	−0.0024 (9)
C19	0.0172 (10)	0.0172 (9)	0.0241 (11)	−0.0030 (8)	0.0005 (9)	−0.0024 (8)
C20	0.0178 (10)	0.0304 (11)	0.0272 (12)	−0.0008 (9)	−0.0006 (9)	−0.0027 (11)
C21	0.0233 (12)	0.0479 (16)	0.0433 (17)	0.0109 (12)	0.0039 (11)	0.0118 (13)
O31	0.0209 (8)	0.0282 (8)	0.0431 (11)	0.0002 (7)	0.0030 (8)	−0.0024 (8)
O52	0.0210 (9)	0.0365 (10)	0.0644 (14)	−0.0070 (8)	0.0033 (9)	−0.0079 (10)
N42	0.0232 (9)	0.0151 (8)	0.0221 (9)	0.0016 (7)	0.0019 (8)	0.0022 (8)
N43	0.0192 (9)	0.0184 (8)	0.0266 (10)	0.0016 (7)	0.0022 (8)	0.0012 (8)
C32	0.0222 (10)	0.0210 (10)	0.0254 (12)	0.0023 (9)	0.0037 (9)	0.0052 (9)
C33	0.0216 (11)	0.0241 (11)	0.0305 (13)	0.0067 (9)	0.0057 (10)	0.0021 (10)
C34	0.0317 (12)	0.0191 (10)	0.0265 (12)	0.0074 (10)	0.0090 (10)	0.0012 (9)
C35	0.0244 (12)	0.0182 (10)	0.0212 (11)	0.0021 (9)	0.0062 (9)	0.0021 (10)
C36	0.0375 (14)	0.0197 (11)	0.0258 (12)	−0.0011 (10)	0.0059 (10)	−0.0031 (10)
C37	0.0320 (13)	0.0283 (12)	0.0310 (14)	−0.0043 (11)	0.0030 (11)	−0.0041 (11)
C38	0.0251 (12)	0.0312 (12)	0.0308 (13)	0.0004 (10)	0.0019 (11)	−0.0018 (11)
C39	0.0214 (11)	0.0221 (11)	0.0279 (12)	0.0020 (9)	0.0032 (10)	−0.0042 (10)
C40	0.0257 (12)	0.0167 (10)	0.0207 (11)	0.0021 (9)	0.0045 (9)	0.0019 (9)
C41	0.0195 (10)	0.0167 (10)	0.0213 (12)	0.0024 (8)	0.0041 (9)	0.0040 (9)
C44	0.0219 (10)	0.0169 (9)	0.0194 (11)	0.0009 (8)	0.0040 (9)	0.0023 (9)
C45	0.0174 (10)	0.0245 (10)	0.0260 (12)	−0.0010 (9)	−0.0006 (9)	0.0005 (9)
C46	0.0226 (11)	0.0239 (11)	0.0266 (12)	−0.0047 (9)	−0.0029 (9)	−0.0033 (10)
C47	0.0246 (12)	0.0204 (10)	0.0257 (12)	0.0014 (9)	0.0029 (10)	−0.0033 (10)
C48	0.0216 (11)	0.0207 (10)	0.0200 (11)	−0.0006 (9)	0.0034 (9)	0.0023 (9)
C49	0.0207 (11)	0.0175 (9)	0.0224 (12)	−0.0025 (8)	0.0019 (9)	0.0001 (9)
C50	0.0204 (12)	0.0296 (12)	0.0274 (13)	0.0010 (10)	0.0045 (10)	−0.0001 (11)
C51	0.0296 (13)	0.0503 (16)	0.0483 (19)	0.0136 (13)	−0.0070 (13)	−0.0217 (14)

### Geometric parameters (Å, º)

**Table:**

O1—C2	1.250 (3)	C16—H16	0.9300
O22—C20	1.214 (3)	C17—H17	0.9300
O31—C32	1.247 (3)	C19—H19	0.9300
O52—C50	1.214 (3)	C21—H21B	0.9600
N12—C11	1.324 (3)	C21—H21C	0.9600
N12—N13	1.314 (3)	C21—H21A	0.9600
N13—C14	1.407 (3)	C32—C33	1.450 (4)
N13—H13	0.8600	C32—C41	1.466 (3)
N42—N43	1.310 (3)	C33—C34	1.337 (4)
N42—C41	1.325 (3)	C34—C35	1.445 (4)
N43—C44	1.412 (3)	C35—C40	1.420 (4)
N43—H43	0.8600	C35—C36	1.400 (4)
C2—C11	1.474 (3)	C36—C37	1.377 (4)
C2—C3	1.455 (4)	C37—C38	1.397 (4)
C3—C4	1.341 (4)	C38—C39	1.371 (4)
C4—C5	1.451 (4)	C39—C40	1.409 (3)
C5—C10	1.413 (3)	C40—C41	1.456 (3)
C5—C6	1.389 (4)	C44—C45	1.392 (3)
C6—C7	1.383 (4)	C44—C49	1.393 (3)
C7—C8	1.394 (4)	C45—C46	1.386 (4)
C8—C9	1.375 (4)	C46—C47	1.395 (4)
C9—C10	1.407 (3)	C47—C48	1.389 (3)
C10—C11	1.453 (3)	C48—C50	1.494 (4)
C14—C19	1.392 (3)	C48—C49	1.396 (3)
C14—C15	1.397 (3)	C50—C51	1.505 (4)
C15—C16	1.379 (4)	C33—H33	0.9300
C16—C17	1.397 (3)	C34—H34	0.9300
C17—C18	1.382 (3)	C36—H36	0.9300
C18—C19	1.402 (3)	C37—H37	0.9300
C18—C20	1.506 (3)	C38—H38	0.9300
C20—C21	1.494 (4)	C39—H39	0.9300
C3—H3	0.9300	C45—H45	0.9300
C4—H4	0.9300	C46—H46	0.9300
C6—H6	0.9300	C47—H47	0.9300
C7—H7	0.9300	C49—H49	0.9300
C8—H8	0.9300	C51—H51A	0.9600
C9—H9	0.9300	C51—H51B	0.9600
C15—H15	0.9300	C51—H51C	0.9600
			
O1···N12	2.854 (4)	C41···C46^vii^	3.575 (5)
O1···N13	2.580 (3)	C41···C44^vii^	3.555 (4)
O1···C20^i^	3.336 (4)	C44···C40^vi^	3.506 (4)
O1···O22^i^	3.165 (4)	C44···C41^vi^	3.555 (4)
O1···C8^ii^	3.398 (4)	C44···C34^vi^	3.541 (4)
O1···C21^i^	3.180 (4)	C44···C35^vi^	3.485 (4)
O22···C15^iii^	3.256 (4)	C45···C32^vi^	3.352 (4)
O22···O1^iii^	3.165 (4)	C45···C41^vi^	3.486 (5)
O31···N42	2.856 (3)	C45···O52^iv^	3.256 (4)
O31···N43	2.575 (3)	C46···C41^vi^	3.575 (5)
O31···C50^iv^	3.360 (4)	C47···N42^vi^	3.357 (4)
O31···C51^iv^	3.165 (5)	C49···C39^vi^	3.582 (5)
O31···O52^iv^	3.180 (4)	C49···C35^vi^	3.584 (5)
O31···C38^i^	3.393 (4)	C49···C40^vi^	3.349 (4)
O52···C45^v^	3.256 (4)	C50···O31^v^	3.360 (4)
O52···O31^v^	3.180 (4)	C51···O31^v^	3.165 (5)
O1···H21A^i^	2.7100	C2···H13	2.4700
O1···H8^ii^	2.7300	C4···H36^viii^	2.7500
O1···H13	1.9100	C5···H36^viii^	2.7400
O22···H19	2.4900	C7···H3^xiii^	3.0100
O22···H15^iii^	2.3600	C8···H3^xiii^	3.1000
O22···H13^iii^	2.8200	C15···H46^vii^	3.0900
O31···H43	1.9000	C15···H47^vii^	3.0900
O31···H38^i^	2.7200	C16···H21A^iv^	3.0800
O31···H51A^iv^	2.7400	C16···H47^vii^	2.9000
O52···H45^v^	2.3600	C17···H21C	2.8900
O52···H21B	2.8600	C17···H21B	2.9100
O52···H49	2.4800	C17···H47^vii^	3.0200
O52···H43^v^	2.8300	C18···H51C^vii^	2.8300
N12···O1	2.854 (4)	C20···H51C^vii^	2.8600
N12···C4^vi^	3.379 (4)	C21···H17	2.6200
N12···C5^vi^	3.380 (4)	C32···H43	2.4600
N12···C17^vii^	3.301 (4)	C34···H6^xi^	2.7900
N12···C6^vi^	3.377 (4)	C35···H6^xi^	2.7600
N12···C16^vii^	3.411 (4)	C37···H33^x^	3.0000
N13···C4^vi^	3.110 (4)	C38···H33^x^	3.0800
N13···C5^vi^	3.384 (4)	C45···H16	3.0300
N13···O1	2.580 (3)	C46···H16	3.0200
N13···C16^vii^	3.448 (4)	C47···H51C	2.8400
N42···C47^vii^	3.357 (4)	C47···H51B	2.9800
N42···O31	2.856 (3)	C48···H17	2.9900
N42···C36^vi^	3.397 (4)	C50···H21B	3.0500
N42···C35^vi^	3.387 (4)	C51···H47	2.6300
N42···C34^vi^	3.382 (4)	H3···C7^xiv^	3.0100
N43···C35^vi^	3.397 (4)	H3···C8^xiv^	3.1000
N43···O31	2.575 (3)	H4···H6	2.4500
N43···C34^vi^	3.118 (4)	H6···H4	2.4500
N12···H9	2.5100	H6···C34^ix^	2.7900
N12···H19	2.4800	H6···C35^ix^	2.7600
N42···H39	2.5000	H8···O1^x^	2.7300
N42···H49	2.4700	H9···N12	2.5100
C2···C15^vii^	3.301 (4)	H13···C2	2.4700
C3···C15^vii^	3.598 (5)	H13···H15	2.3900
C4···N13^vii^	3.110 (4)	H13···O22^i^	2.8200
C4···C14^vii^	3.528 (5)	H13···O1	1.9100
C4···N12^vii^	3.379 (4)	H15···H13	2.3900
C4···C36^viii^	3.550 (5)	H15···O22^i^	2.3600
C5···N13^vii^	3.384 (4)	H16···C46	3.0200
C5···N12^vii^	3.380 (4)	H16···H21A^iv^	2.4200
C5···C36^viii^	3.577 (5)	H16···C45	3.0300
C5···C14^vii^	3.466 (4)	H17···C21	2.6200
C5···C19^vii^	3.596 (4)	H17···C48	2.9900
C6···C35^ix^	3.595 (5)	H17···H21B	2.4000
C6···N12^vii^	3.377 (4)	H17···H21C	2.4500
C6···C34^ix^	3.578 (5)	H19···O22	2.4900
C8···O1^x^	3.398 (4)	H19···N12	2.4800
C9···C19^vii^	3.588 (5)	H21A···O1^iii^	2.7100
C10···C19^vii^	3.360 (4)	H21A···H16^v^	2.4200
C10···C14^vii^	3.486 (4)	H21A···C16^v^	3.0800
C11···C14^vii^	3.541 (5)	H21B···C17	2.9100
C11···C16^vii^	3.513 (5)	H21B···C50	3.0500
C11···C15^vii^	3.436 (4)	H21B···O52	2.8600
C14···C4^vi^	3.528 (5)	H21B···H17	2.4000
C14···C5^vi^	3.466 (4)	H21C···C17	2.8900
C14···C11^vi^	3.541 (5)	H21C···H17	2.4500
C14···C10^vi^	3.486 (4)	H33···C37^ii^	3.0000
C15···C3^vi^	3.598 (5)	H33···C38^ii^	3.0800
C15···O22^i^	3.256 (4)	H34···H36	2.4700
C15···C2^vi^	3.301 (4)	H36···H34	2.4700
C15···C11^vi^	3.436 (4)	H36···C4^xii^	2.7500
C16···N13^vi^	3.448 (4)	H36···C5^xii^	2.7400
C16···C11^vi^	3.513 (5)	H38···O31^iii^	2.7200
C16···N12^vi^	3.411 (4)	H39···N42	2.5000
C17···N12^vi^	3.301 (4)	H43···O31	1.9000
C19···C5^vi^	3.596 (4)	H43···C32	2.4600
C19···C10^vi^	3.360 (4)	H43···H45	2.3900
C19···C9^vi^	3.588 (5)	H43···O52^iv^	2.8300
C20···O1^iii^	3.336 (4)	H45···H43	2.3900
C21···O1^iii^	3.180 (4)	H45···O52^iv^	2.3600
C32···C45^vii^	3.352 (4)	H46···C15^vi^	3.0900
C34···N42^vii^	3.382 (4)	H46···H51A^xv^	2.4800
C34···C6^xi^	3.578 (5)	H47···C15^vi^	3.0900
C34···N43^vii^	3.118 (4)	H47···C16^vi^	2.9000
C34···C44^vii^	3.541 (4)	H47···C17^vi^	3.0200
C35···C44^vii^	3.485 (4)	H47···C51	2.6300
C35···C6^xi^	3.595 (5)	H47···H51B	2.5700
C35···N43^vii^	3.397 (4)	H47···H51C	2.3100
C35···N42^vii^	3.387 (4)	H49···O52	2.4800
C35···C49^vii^	3.584 (5)	H49···N42	2.4700
C36···N42^vii^	3.397 (4)	H51A···O31^v^	2.7400
C36···C5^xii^	3.577 (5)	H51A···H46^xvi^	2.4800
C36···C4^xii^	3.550 (5)	H51B···C47	2.9800
C38···O31^iii^	3.393 (4)	H51B···H47	2.5700
C39···C49^vii^	3.582 (5)	H51C···C18^vi^	2.8300
C40···C49^vii^	3.349 (4)	H51C···C20^vi^	2.8600
C40···C44^vii^	3.506 (4)	H51C···C47	2.8400
C41···C45^vii^	3.486 (5)	H51C···H47	2.3100
			
N13—N12—C11	120.50 (19)	H21A—C21—H21B	110.00
N12—N13—C14	119.17 (18)	C20—C21—H21A	109.00
N12—N13—H13	120.00	C20—C21—H21B	109.00
C14—N13—H13	120.00	C20—C21—H21C	109.00
N43—N42—C41	119.53 (18)	C33—C32—C41	117.1 (2)
N42—N43—C44	118.67 (17)	O31—C32—C41	121.1 (2)
N42—N43—H43	121.00	O31—C32—C33	121.7 (2)
C44—N43—H43	121.00	C32—C33—C34	121.8 (2)
O1—C2—C11	121.5 (2)	C33—C34—C35	122.8 (2)
O1—C2—C3	121.2 (2)	C34—C35—C36	121.5 (2)
C3—C2—C11	117.3 (2)	C34—C35—C40	119.0 (2)
C2—C3—C4	121.1 (2)	C36—C35—C40	119.6 (2)
C3—C4—C5	123.1 (2)	C35—C36—C37	120.9 (2)
C4—C5—C6	121.0 (2)	C36—C37—C38	119.7 (2)
C6—C5—C10	119.9 (2)	C37—C38—C39	120.5 (2)
C4—C5—C10	119.2 (2)	C38—C39—C40	121.3 (2)
C5—C6—C7	121.0 (2)	C35—C40—C39	118.0 (2)
C6—C7—C8	119.2 (2)	C35—C40—C41	119.1 (2)
C7—C8—C9	121.0 (2)	C39—C40—C41	122.9 (2)
C8—C9—C10	120.4 (2)	N42—C41—C32	124.1 (2)
C5—C10—C9	118.5 (2)	C32—C41—C40	120.1 (2)
C5—C10—C11	119.1 (2)	N42—C41—C40	115.7 (2)
C9—C10—C11	122.4 (2)	N43—C44—C49	121.1 (2)
C2—C11—C10	120.2 (2)	C45—C44—C49	120.8 (2)
N12—C11—C2	123.2 (2)	N43—C44—C45	118.1 (2)
N12—C11—C10	116.5 (2)	C44—C45—C46	119.4 (2)
C15—C14—C19	120.7 (2)	C45—C46—C47	120.6 (2)
N13—C14—C19	121.1 (2)	C46—C47—C48	119.5 (2)
N13—C14—C15	118.24 (19)	C49—C48—C50	117.5 (2)
C14—C15—C16	119.6 (2)	C47—C48—C49	120.5 (2)
C15—C16—C17	120.3 (2)	C47—C48—C50	122.0 (2)
C16—C17—C18	120.2 (2)	C44—C49—C48	119.1 (2)
C19—C18—C20	117.8 (2)	C48—C50—C51	118.9 (2)
C17—C18—C20	122.1 (2)	O52—C50—C48	120.4 (2)
C17—C18—C19	120.1 (2)	O52—C50—C51	120.7 (2)
C14—C19—C18	119.1 (2)	C32—C33—H33	119.00
C18—C20—C21	118.5 (2)	C34—C33—H33	119.00
O22—C20—C18	120.0 (2)	C33—C34—H34	119.00
O22—C20—C21	121.5 (2)	C35—C34—H34	119.00
C4—C3—H3	119.00	C35—C36—H36	119.00
C2—C3—H3	120.00	C37—C36—H36	120.00
C3—C4—H4	118.00	C36—C37—H37	120.00
C5—C4—H4	118.00	C38—C37—H37	120.00
C5—C6—H6	120.00	C37—C38—H38	120.00
C7—C6—H6	119.00	C39—C38—H38	120.00
C6—C7—H7	120.00	C38—C39—H39	119.00
C8—C7—H7	120.00	C40—C39—H39	119.00
C9—C8—H8	120.00	C44—C45—H45	120.00
C7—C8—H8	119.00	C46—C45—H45	120.00
C8—C9—H9	120.00	C45—C46—H46	120.00
C10—C9—H9	120.00	C47—C46—H46	120.00
C16—C15—H15	120.00	C46—C47—H47	120.00
C14—C15—H15	120.00	C48—C47—H47	120.00
C17—C16—H16	120.00	C44—C49—H49	120.00
C15—C16—H16	120.00	C48—C49—H49	120.00
C18—C17—H17	120.00	C50—C51—H51A	109.00
C16—C17—H17	120.00	C50—C51—H51B	109.00
C14—C19—H19	120.00	C50—C51—H51C	109.00
C18—C19—H19	120.00	H51A—C51—H51B	109.00
H21A—C21—H21C	109.00	H51A—C51—H51C	109.00
H21B—C21—H21C	109.00	H51B—C51—H51C	109.00
			
C11—N12—N13—C14	179.7 (2)	C19—C18—C20—C21	171.1 (2)
N13—N12—C11—C2	−1.2 (3)	C17—C18—C19—C14	0.7 (4)
N13—N12—C11—C10	−178.94 (19)	C20—C18—C19—C14	179.8 (2)
N12—N13—C14—C15	177.8 (2)	C17—C18—C20—O22	169.1 (2)
N12—N13—C14—C19	−1.3 (3)	C17—C18—C20—C21	−9.9 (4)
C41—N42—N43—C44	−179.4 (2)	O31—C32—C33—C34	−178.2 (2)
N43—N42—C41—C32	1.3 (3)	C41—C32—C33—C34	2.7 (3)
N43—N42—C41—C40	179.2 (2)	O31—C32—C41—N42	−6.0 (4)
N42—N43—C44—C45	−179.7 (2)	O31—C32—C41—C40	176.2 (2)
N42—N43—C44—C49	−0.5 (3)	C33—C32—C41—N42	173.1 (2)
C3—C2—C11—C10	4.6 (3)	C33—C32—C41—C40	−4.7 (3)
O1—C2—C11—N12	5.5 (4)	C32—C33—C34—C35	−0.1 (4)
O1—C2—C3—C4	178.7 (2)	C33—C34—C35—C36	179.3 (2)
C11—C2—C3—C4	−2.7 (4)	C33—C34—C35—C40	−0.5 (4)
O1—C2—C11—C10	−176.8 (2)	C34—C35—C36—C37	−179.8 (2)
C3—C2—C11—N12	−173.1 (2)	C40—C35—C36—C37	0.1 (4)
C2—C3—C4—C5	0.1 (4)	C34—C35—C40—C39	−179.8 (2)
C3—C4—C5—C6	−179.3 (2)	C34—C35—C40—C41	−1.5 (3)
C3—C4—C5—C10	0.7 (4)	C36—C35—C40—C39	0.4 (3)
C4—C5—C10—C11	1.3 (3)	C36—C35—C40—C41	178.7 (2)
C4—C5—C6—C7	179.8 (2)	C35—C36—C37—C38	−0.2 (4)
C6—C5—C10—C9	−0.4 (3)	C36—C37—C38—C39	0.0 (4)
C6—C5—C10—C11	−178.8 (2)	C37—C38—C39—C40	0.5 (4)
C10—C5—C6—C7	−0.1 (3)	C38—C39—C40—C35	−0.7 (4)
C4—C5—C10—C9	179.7 (2)	C38—C39—C40—C41	−178.9 (2)
C5—C6—C7—C8	0.4 (4)	C35—C40—C41—N42	−173.8 (2)
C6—C7—C8—C9	−0.2 (4)	C35—C40—C41—C32	4.2 (3)
C7—C8—C9—C10	−0.3 (4)	C39—C40—C41—N42	4.4 (3)
C8—C9—C10—C5	0.6 (3)	C39—C40—C41—C32	−177.7 (2)
C8—C9—C10—C11	179.0 (2)	N43—C44—C45—C46	179.2 (2)
C5—C10—C11—C2	−3.9 (3)	C49—C44—C45—C46	0.0 (3)
C9—C10—C11—N12	−4.5 (3)	N43—C44—C49—C48	−178.2 (2)
C5—C10—C11—N12	173.9 (2)	C45—C44—C49—C48	1.0 (3)
C9—C10—C11—C2	177.7 (2)	C44—C45—C46—C47	−0.7 (4)
N13—C14—C15—C16	−178.9 (2)	C45—C46—C47—C48	0.4 (4)
C19—C14—C15—C16	0.3 (4)	C46—C47—C48—C49	0.6 (3)
C15—C14—C19—C18	−1.3 (4)	C46—C47—C48—C50	179.1 (2)
N13—C14—C19—C18	177.9 (2)	C47—C48—C49—C44	−1.3 (3)
C14—C15—C16—C17	1.3 (4)	C50—C48—C49—C44	−179.9 (2)
C15—C16—C17—C18	−1.9 (4)	C47—C48—C50—O52	−167.2 (3)
C16—C17—C18—C19	0.9 (4)	C47—C48—C50—C51	11.7 (4)
C16—C17—C18—C20	−178.2 (2)	C49—C48—C50—O52	11.3 (4)
C19—C18—C20—O22	−10.0 (4)	C49—C48—C50—C51	−169.8 (2)

Symmetry codes: (i) *x*+1/2, −*y*+1, *z*; (ii) *x*+1/2, −*y*, *z*; (iii) *x*−1/2, −*y*+1, *z*; (iv) *x*+1/2, −*y*+2, *z*; (v) *x*−1/2, −*y*+2, *z*; (vi) *x*, *y*+1, *z*; (vii) *x*, *y*−1, *z*; (viii) −*x*+3/2, *y*, *z*+1/2; (ix) −*x*+3/2, *y*−1, *z*+1/2; (x) *x*−1/2, −*y*, *z*; (xi) −*x*+3/2, *y*+1, *z*−1/2; (xii) −*x*+3/2, *y*, *z*−1/2; (xiii) *x*−1/2, −*y*−1, *z*; (xiv) *x*+1/2, −*y*−1, *z*; (xv) *x*+1/2, −*y*+3, *z*; (xvi) *x*−1/2, −*y*+3, *z*.

### Hydrogen-bond geometry (Å, º)

**Table:**

*D*—H···*A*	*D*—H	H···*A*	*D*···*A*	*D*—H···*A*
N13—H13···O1	0.86	1.91	2.580 (3)	134
N43—H43···O31	0.86	1.90	2.575 (3)	134
C15—H15···O22^i^	0.93	2.36	3.256 (4)	162
C45—H45···O52^iv^	0.93	2.36	3.256 (4)	162

Symmetry codes: (i) *x*+1/2, −*y*+1, *z*; (iv) *x*+1/2, −*y*+2, *z*.
